# Supplementary material for: Optimization of the proliferation and persistency of CAR T cells derived from human induced pluripotent stem cells
Source: Nat Biomed Eng. 2022 Dec 12;7(1):24–37. doi: 10.1038/s41551-022-00969-0 (PMC9870784; doi:10.1038/s41551-022-00969-0)
Supplement: Supplementary file 1 — Supplementary figures and tables. [file 41551_2022_969_MOESM1_ESM.pdf]

nature biomedical engineering

Article https://doi.org/10.1038/s41551-022-00969-0

Optimization of the proliferation and persistency of CAR T cells derived from human induced pluripotent stem cells

In the format provided by the authors and unedited

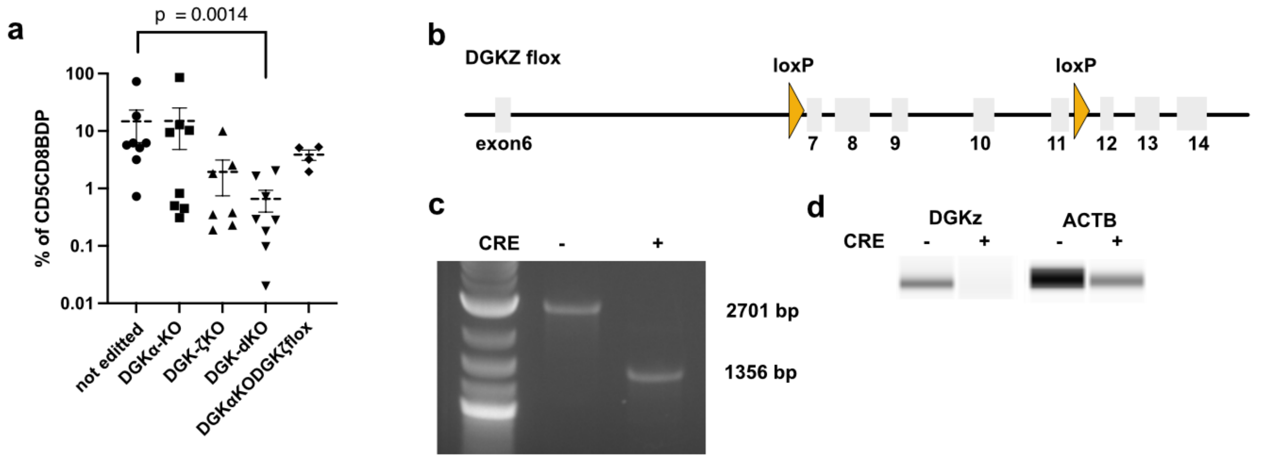

Supplementary Fig. 1 | The strategy to produce DGK-dKO-iCAR-T_{CTL} efficiently using DGKα-/-DGKζf/f iPSCs

a. Ratio of CD5CD8β DP cells to live differentiated cells derived from wild-type iCAR, DGKa-KO iCAR, DGKz-KO iCAR, DGK-dKO iCAR, and DGKa$^{-/-}$DGKz$^{f/f}$ CAR-iPSCs (n=4-8 mean ± SEM). One-way ANOVA with Tukey's multiple comparisons test. **b.** Scheme of the DGK flox sequence. To create DGK-dKO-iCAR-T_{CTL} loxP sequence was inserted between exon 6 and exon 7 and exon 11 and exon 12. **c.** DGKz flox iPSCs were differentiated into T cells. Differentiated T cells were transduced with CRE with the retrovirus. The DNA of CRE-transduced iCAR-TCTL derived from DGKα-/-DGKζf/f iPSCs was extracted and the sequence including the floxed region was amplified to confirm the deletion **d.** Protein was extracted from both CRE transduced and non-transduced iCAR-TCTL derived from DGKα-/-DGKζf/f iPSCs and evaluated for the disruption of DGKz by western blotting.

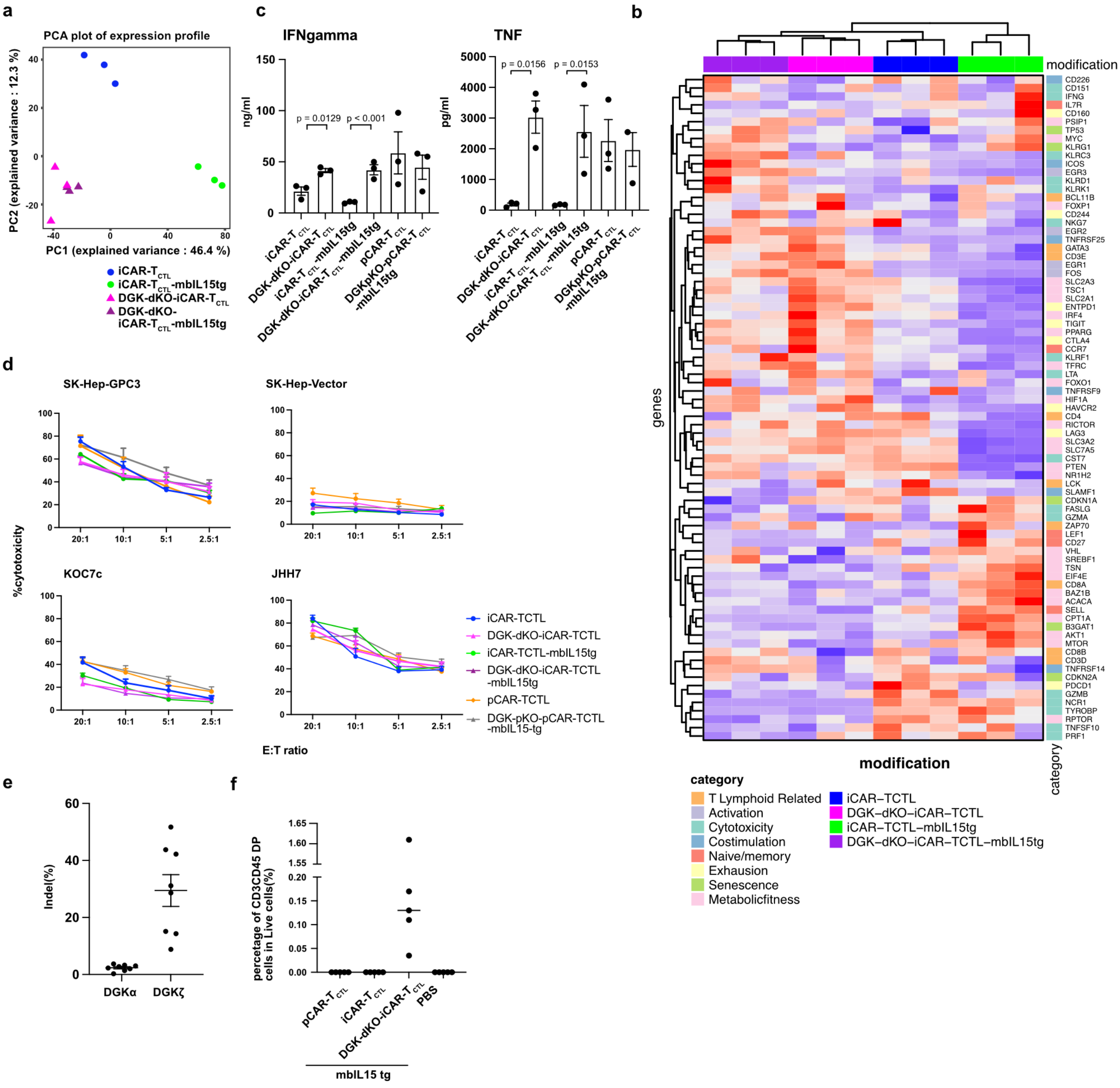

Supplementary Fig. 2 | Combinatorial signal-enhanced iCAR-T_{CTL} gene expression profile and anti-tumor function *in vitro*

a. A PCA of global transcriptional profiles of iCAR T cells (iCAR-T_{CTL}, iCAR-T_{CTL}-mbIL15tg, DGK-dKO-iCAR-T_{CTL}, and DGK-dKO- iCAR-T_{CTL}-mbIL15tg). **b.** Heat map of T lymphoid, activation, cytotoxicity, co-stimulation, naïve/memory, exhausion, senescence- and metabolic fitness-related genes[52,53,54]. Shown are cpm values. **c.** Cytokine production of iCAR-T_{CTL}, DGK-dKO-iCAR-T_{CTL}, iCAR-T_{CTL}-mbIL15tg, DGK-dKO-iCAR-T_{CTL}-mbIL15tg pCAR-T_{CTL}, and DGK-pKO- pCAR-T_{CTL}-mbIL15tg 48 h after co-culturing with irradiated SK-Hep-GPC3 (n = 3, mean ± SEM). One-way ANOVA with Tukey's multiple comparisons test. **d.** ^{51}Cr release assay of iCAR-T_{CTL}, DGK-dKO-iCAR-T_{CTL}, iCAR-T_{CTL}-mbIL15tg, DGK-dKO-iCAR-T_{CTL}-mbIL15tg, pCAR-T_{CTL}, and DGK-pKO-pCAR-T_{CTL}-mbIL15tg against GPC3 positive (SK-Hep-GPC3, Koc7c, and JHH7) or negative (SK-Hep-Vector) cancer cell lines (n=3 mean ± SEM). **e.** Knockout efficiency of DGK genes in primary T cells determined by tracking of indels by decomposition (TIDE) analysis (n = 8 mean ± SEM). **f.** Percentage of CD3CD45 DP human T cells in the peripheral blood cells of tumor-bearing mice 28 days after the treatment. Values were determined by flow cytometry (n = 5 per cohort).

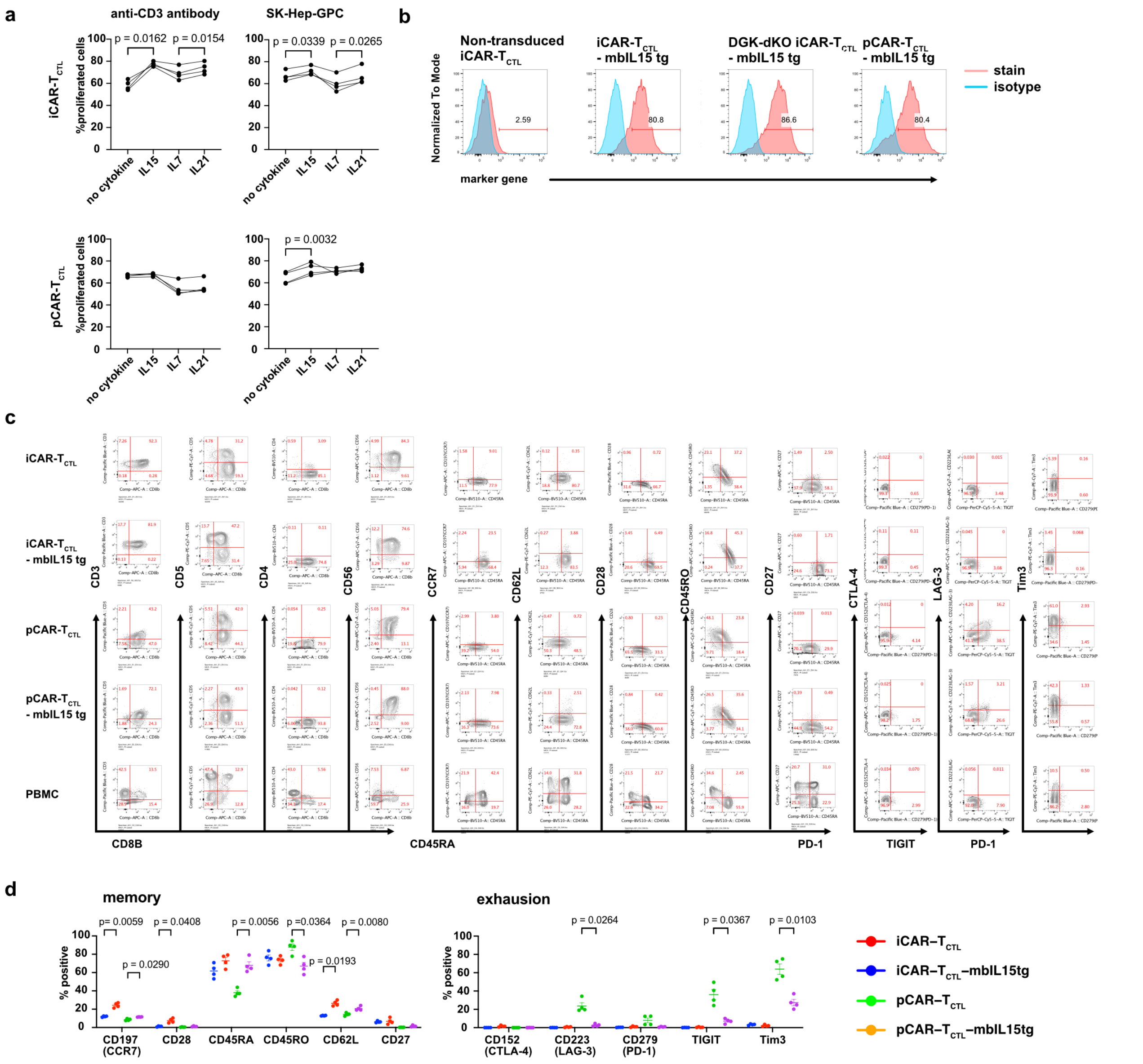

Supplementary Fig. 3 | Signal 3 enhancement with mbIL15 increased the memory marker expression and decreased the exhaustion marker expression

a. Enhancement of proliferation capability of iCAR-T_{CTL} or pCAR-T_{CTL} with additional signal 3-related cytokines. Fluorescence-labeled iCAR-T_{CTL} or pCAR-T_{CTL} is stimulated with irradiated SK-Hep-GPC3. Each of indicated cytokines is added to the culture medium. One-way ANOVA with Tukey's multiple comparisons test. **b.** Expression of tNGFR in mbIL-15-transduced iCAR-T_{CTL}, DGK-dKO-iCAR-T_{CTL}, and pCAR-T_{CTL}. **c-d.** Surface antigen profiles of iCAR-T_{CTL}, iCAR-T_{CTL}-mbIL15tg, pCAR-T_{CTL} and pCAR-T_{CTL}-mbIL15tg. iCAR-T_{CTL}, iCAR-T_{CTL}-mbIL15tg, pCAR-T_{CTL} and pCAR-T_{CTL}-mbIL15tg were evaluated using surface antigen expression related to naïve/memory, and exhaustion phenotype. One-way ANOVA with Tukey's multiple comparisons test.

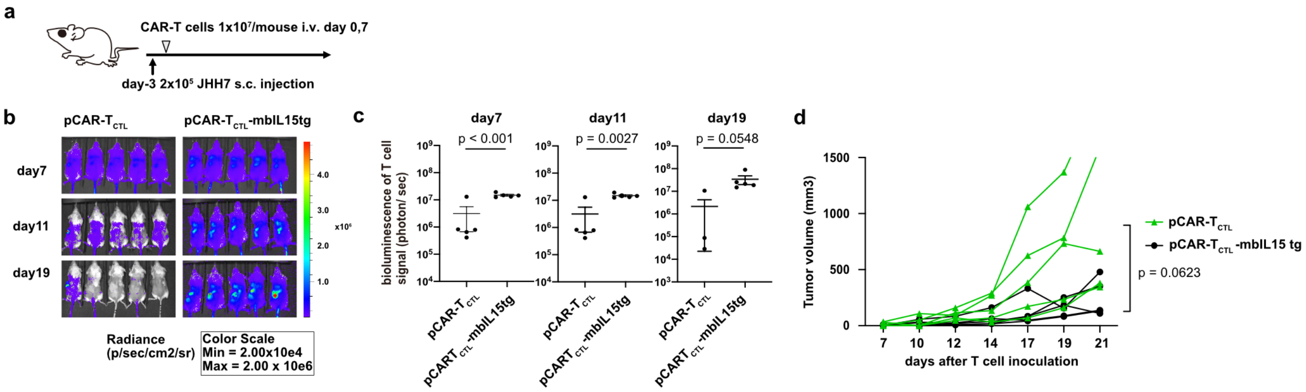

Supplementary Fig. 4 l Signal enhancement improved the accumulation, persistency, and effector function of pCAR-T$_{CTL}$

a-d. Treatment schedule of the liver cancer subcutaneous xenograft model (**a**). NSG mice were injected subcutaneously with 2 × 10^5 JHH7 cells 3 days before the treatment. Three days after the liver cancer inoculation, 1 × 10^7 pCAR-T$_{CTL}$ or pCAR-T$_{CTL}$-mbIL15tg were administered intravenously (*n* = 5 for each group, mean ± SEM). *In vivo* bioluminescence imaging of injected T cells in NSG mice (**b**). Total flux (photons/s) of injected pCAR-T cells in the JHH7 tumor was quantified at the indicated time points (**c**). Student's *t*-test. Tumor volume of inoculated JHH7 at the indicated time points in individual mice treated with the indicated test cells. Mean tumor size ± SEM (*n* = 5 of each group). Two-way ANOVA **(d)**.

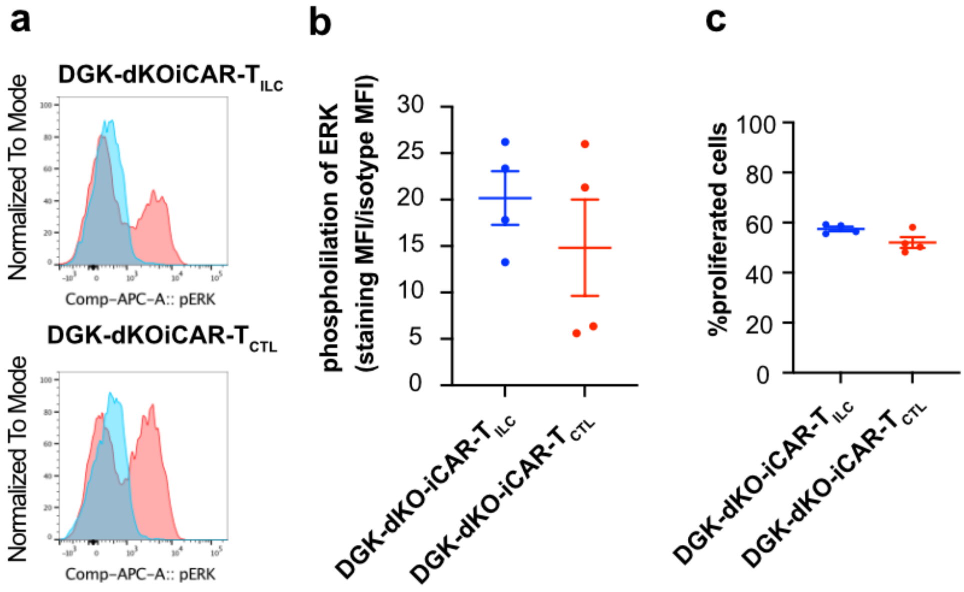

Supplementary Fig.5 | DGK-dKO-iCAR-T$_{ILC}$ have comparable tumor suppressive function DGK-dKO-iCAR-T$_{CTL}$

a-b Detection of phosphorylated ERK (pERK) in DGKdKO-iCAR-T$_{CTL}$ and DGKdKO-iCAR-TILC 60 min after co-culturing with irradiated SK-Hep-GPC3. (n=4 mean ± SEM). **c.** Proliferation assay of DGKdKO-iCAR-T$_{CTL}$ and DGKdKO-iCAR-T$_{ILC}$ *n* = 4, mean ± SEM. SEM, standard error of the mean (n=4 mean ± SEM).

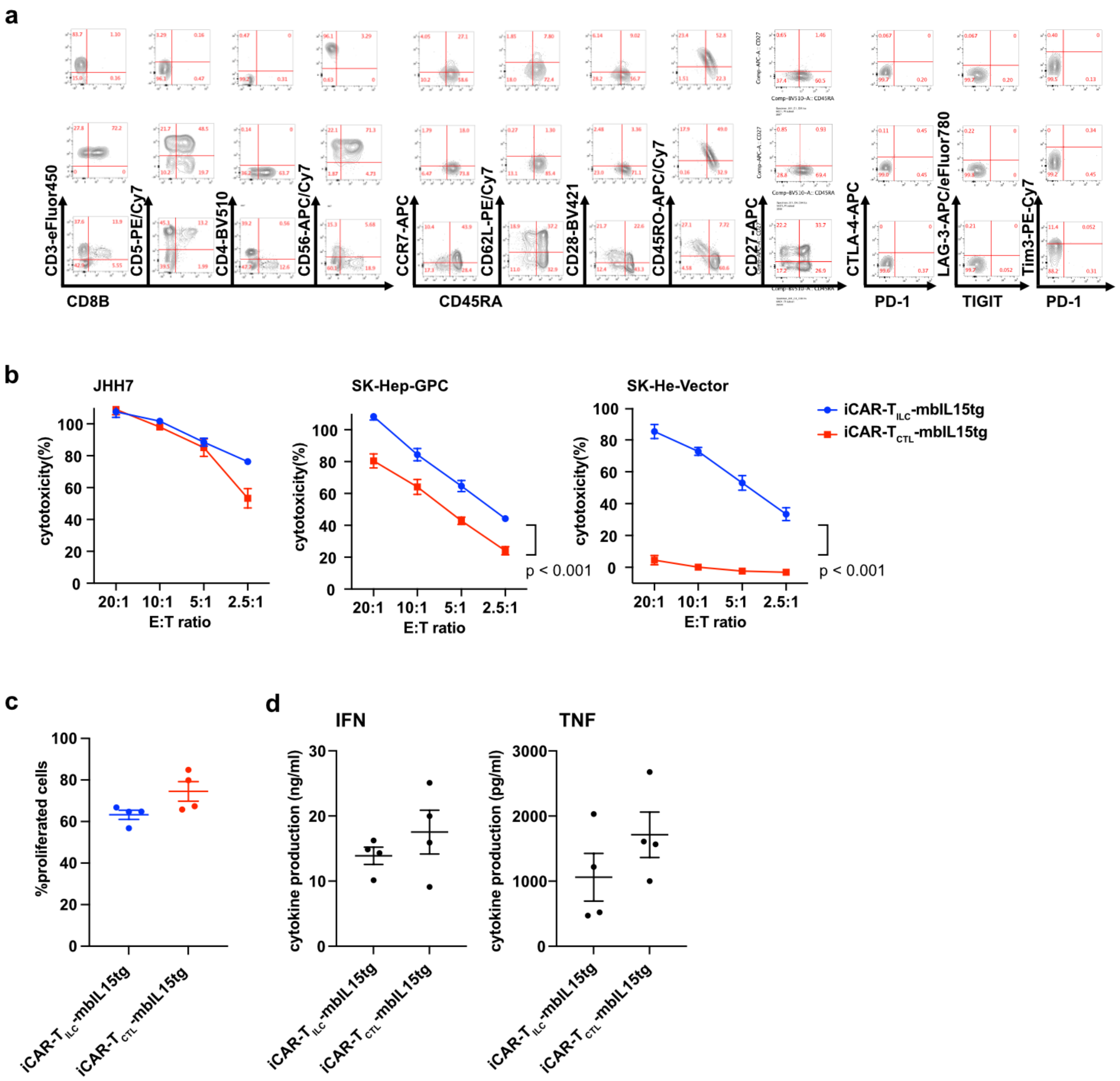

Supplementary Fig. 6 I iCAR-T$_{ILC}$-mbIL15tg have a comparable function to iCAR-T$_{CTL}$-mbIL15tg *in vitro*

a Surface antigen profiles of dKO-iCAR-T$_{CTL}$-mbIL15tg and iCAR-T$_{ILC}$-mbIL15tg. dKO-iCAR-T$_{CTL}$-mbIL15tg and iCAR-T$_{ILC}$-mbIL15tg were evaluated using surface antigen expression related to T/NK linage, naïve/memory, and exhaustion phenotype. **b**. *In vitro* cytotoxic assays of dKO-iCAR-T$_{CTL}$-mbIL15tg and iCAR-T$_{ILC}$-mbIL15tg against JHH7, SK-Hep-GPC3, and SK-Hep-Vector (n = 4, mean ± SEM). Two-way ANOVA. **c.** Proliferation assay of dKO-iCAR-T$_{CTL}$-mbIL15tg and iCAR-T$_{ILC}$-mbIL15tg (n=4 mean ± SEM). **d.** Cytokine production dKO-iCAR-T$_{CTL}$-mbIL15tg and iCAR-T$_{ILC}$-mbIL15tg 48 h after coculturing with irradiated SK-Hep-GPC3 (n= 4) (n=4 mean ± SEM).

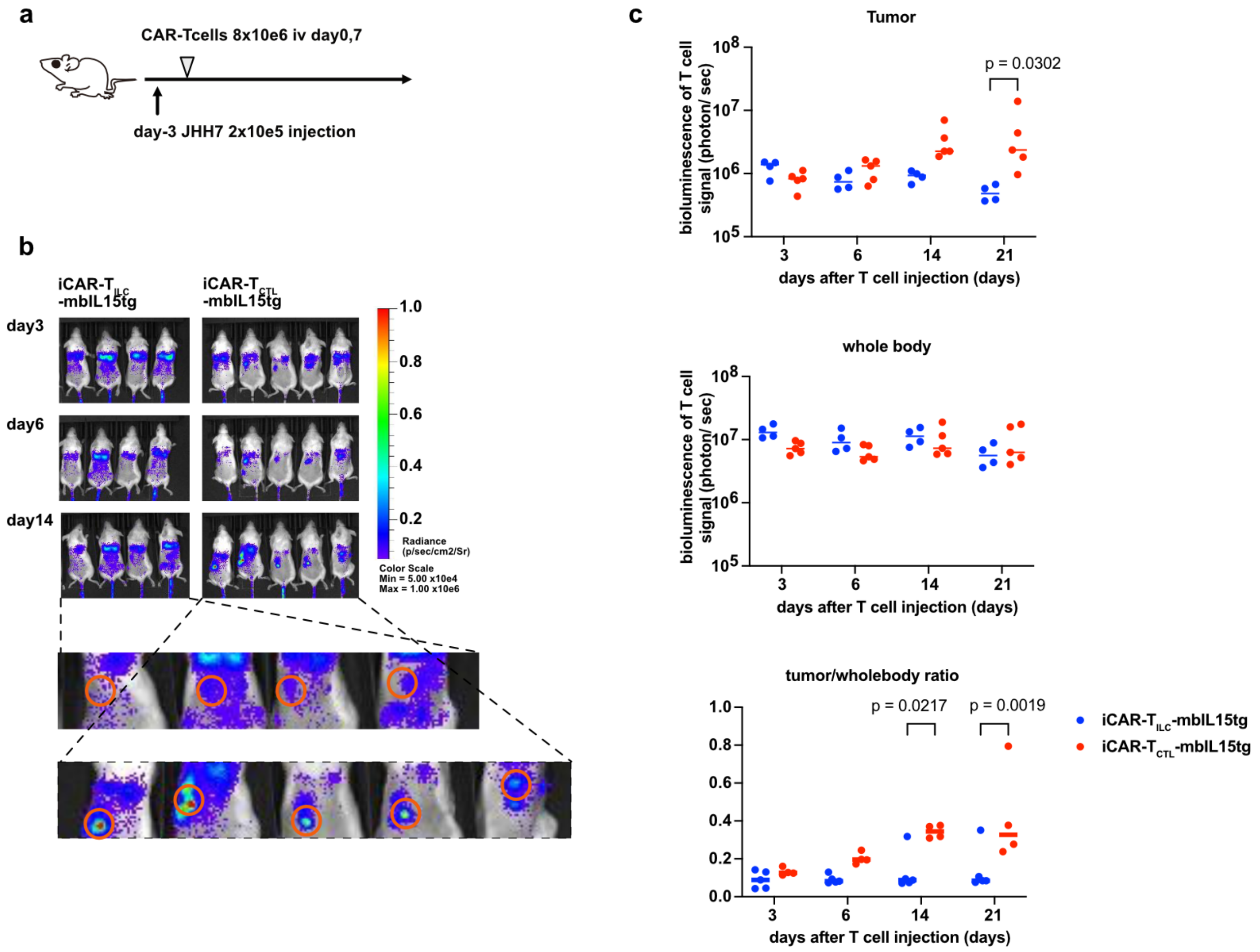

Supplementary Fig. 7 | iCAR-T$_{ILC}$-mbIL15tg showed different distribution *in vivo* from iCAR-T$_{CTL}$-mbIL15tg

a-c. NSG mice were injected subcutaneously with 2×10^5 JHH7 cells 3 days before the treatment. 8×10^6 dKO-iCAR-T$_{CTL}$-mbIL15tg and iCAR-T$_{ILC}$-mbIL15tg were injected intravenously on days 0 and 7 **(a)**. *In vivo* bioluminescence imaging of the injected T cells in NSG mice **(b)**. Total flux (photons/s) from the injected iCAR-T cells in the JHH7 tumor in the tumor (top panel), in the whole body (middle panel) was quantified at the indicated time points (*n* = 4-5 of each group). Student's *t*-test. The bottom panel shows the ratio between tumor site/whole body of T cell signal (*n* = 4-5 mean ± SEM) **(c)**.

a

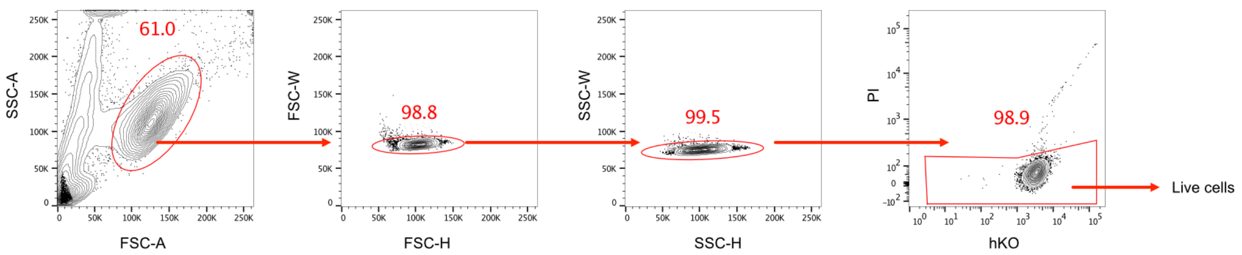

Supplementary Fig. 8 | Flow-cytometry gating strategy.
a. Gating strategy applied to identify live cells.

Supplementary Table 1 | List of genes for the single cell analysis (BD Bioscience Human T-Cell Expression Panel)

gene panel list									
AIM2	CCR2	CD7	DUSP2	GZMA	IL12RB1	IL7R	LAT2	PTTG2	TLR9
ANXA5	CCR3	CD70	DUSP4	GZMB	IL12RB2	IL9	LCK	PYCR1	TNF
APOBEC3G	CCR4	CD8A	EGR1	GZMH	IL13	IL9R	LEF1	RORA	TNFRSF18
ARL4C	CCR5	CD8B	EGR3	GZMK	IL15	IRF4	LGALS1	RORC	TNFRSF1B
AURKB	CCR6	CD9	ENTPD1	GZMM	IL15RA	IRF8	LGALS3	RUNX3	TNFRSF25
B3GAT1	CCR7	CHI3L2	EOMES	HAVCR2	IL17A	ITGA4	LIF	S1PR1	TNFRSF4
BAX	CCR8	CLC	F5	HLA-A	IL17F	ITGAE	LILRB4	SELL	TNFRSF8
BCL11B	CCR9	CLEC2D	FAS	HLA-C	IL18	ITGAL	LRRC32	SELPLG	TNFRSF9
BCL2	CD160	CNOT2	FASLG	HLA-DMA	IL18R1	ITGAM	LTA	SEMA7A	TNFSF10
BCL6	CD2	CSF2	FBXO22	HLA-DMB	IL18RAP	ITGAX	LTB	SLAMF1	TOP2A
BIN2	CD244	CSF3	FOSB	HLA-DPA1	IL1R2	ITGB2	MKI67	SPOCK2	TRAC
BTG1	CD247	CST7	FOSL1	HLA-DPB1	IL2	ITK	MYC	SPP1	TRAT1
BTLA	CD27	CTLA4	FOXO1	HLA-DQA1	IL21	JUN	NAMPT	STAT1	TRBC2
C10orf54	CD274	CTSW	FOXO3	HLA-DQB1	IL22	JUNB	NCR3	STAT3	TRDC
CASP3	CD300A	CX3CR1	FOXP1	HLA-DRA	IL23R	KIT	NINJ2	STAT4	TRIB2
CBLB	CD3D	CXCL10	FOXP3	HLA-DRB3	IL25	KLRB1	NKG7	STAT5A	TSPAN32
CCL1	CD3E	CXCL13	FYB	HMGB2	IL2RA	KLRC1	NT5E	STAT6	TXK
CCL2	CD3G	CXCL8	FYN	HMMR	IL2RB	KLRC3	OAS1	TARP	TYMS
CCL20	CD4	CXCL9	GAPDH	ICAM1	IL3	KLRC4	PASK	TBX21	UBE2C
CCL3	CD40LG	CXCR1	GATA3	ICOS	IL31	KLRF1	PDCD1	TCF7	VNN2
CCL4	CD44	CXCR3	GHR	IER3	IL32	KLRG1	PECAM1	TGFB1	XCL1
CCL5	CD48	CXCR4	GIMAP2	IER5	IL4	KLRK1	PIK3IP1	TGFB3	ZAP70
CCNB1	CD5	CXCR5	GIMAP5	IFNG	IL4R	LAG3	PMCH	TIAF1	ZBED2
CCND2	CD52	CXCR6	GIMAP7	IFNGR1	IL5	LAIR2	PRDM1	TIGIT	ZBTB16
CCR1	CD6	DPP4	GLG1	IKZF2	IL6	LAP3	PRF1	TK1	ZNF683
CCR10	CD69	DUSP1	GNLY	IL12A	IL6R	LAT	PTGDR2	TLR2	

Supplementary Table 2 | GO analysis of DEGs in iCAR-T_{CTL}-mbIL15tg versus iCAR-T_{CTL} and DGK-dKO-iCAR-T_{CTL} versus iCAR-T_{CTL}

iCAR-TCTLmbIL15 vs iCAR-TCTL						
	up-regulated		down-regulated		combined	
**Description**	**Count**	**p-value**	**Count**	**p-value**	**p-value**	**adjusted p-value**
DNA conformation change	75	4.49E-33	2	0.999999275	3.39E-31	4.70E-27
DNA replication	69	7.67E-31	8	0.983544045	5.31E-29	2.56E-25
chromosome organization	149	7.89E-31	28	0.99999985	5.54E-29	2.56E-25
DNA metabolic process	134	3.00E-30	25	0.999995534	2.07E-28	7.17E-25
DNA-dependent DNA replication	51	1.50E-28	4	0.975827777	9.54E-27	2.65E-23
cell cycle	172	1.81E-25	60	0.999437821	1.05E-23	2.43E-20
mitotic cell cycle	123	7.81E-25	33	0.998038453	4.40E-23	8.73E-20
nucleosome assembly	41	6.90E-24	1	0.998995292	3.75E-22	6.50E-19
DNA repair	89	1.86E-23	11	0.999992476	9.95E-22	1.53E-18
protein-DNA complex assembly	54	5.27E-23	2	0.999958876	2.76E-21	3.51E-18

DGKdKO-iCAR-TCTL vs iCAR-TCTL						
	up-regulated		down-regulated		combined	
**Description**	**Count**	**p-value**	**Count**	**p-value**	**p-value**	**adjusted p-value**
regulation of multicellular organismal process	61	2.85E-11	40	8.91E-05	8.80E-14	1.22E-09
inflammatory response	23	1.01E-08	18	1.28E-06	4.27E-13	2.96E-09
locomotion	37	3.53E-08	29	5.06E-06	5.43E-12	2.51E-08
cell migration	29	2.50E-06	26	2.51E-06	1.68E-10	4.77E-07
regulation of cell proliferation	37	1.49E-08	24	0.00043007	1.72E-10	4.77E-07
positive regulation of developmental process	33	1.06E-08	20	0.000970389	2.70E-10	6.10E-07
cell motility	30	4.09E-06	27	3.41E-06	3.63E-10	6.10E-07
localization of cell	30	4.09E-06	27	3.41E-06	3.63E-10	6.10E-07
regulation of response to external stimulus	24	3.75E-08	15	0.00044868	4.35E-10	6.10E-07
cell communication	84	3.47E-07	67	5.42E-05	4.83E-10	6.10E-07

adjusted p-value: Benjamini-Hochberg method

Supplementary Table 3 I Schematic explanation of the effect of DGK-dKO and mbIL15 transduction on iCAR-T_{CTL}

	Cytokine Production	Proliferation	in vivo persistence
DGK-dKO	+	+	+
mbIL15tg	no change	+	++
DGK-dKO + mbIL15tg	+	N/A	+++
