# Supplementary material for: Optimization of the proliferation and persistency of CAR T cells derived from human induced pluripotent stem cells
Source: Nat Biomed Eng. 2022 Dec 12;7(1):24–37. doi: 10.1038/s41551-022-00969-0 (PMC9870784; doi:10.1038/s41551-022-00969-0)
Supplement: Supplementary file 2 — Reporting Summary [file 41551_2022_969_MOESM2_ESM.pdf]

Corresponding author(s): Shin Kaneko

Last updated by author(s): Oct 20, 2022

Reporting Summary

Nature Portfolio wishes to improve the reproducibility of the work that we publish. This form provides structure for consistency and transparency in reporting. For further information on Nature Portfolio policies, see our Editorial Policies and the Editorial Policy Checklist.

Statistics

For all statistical analyses, confirm that the following items are present in the figure legend, table legend, main text, or Methods section.

n/a	Confirmed	
☐	☒	The exact sample size (*n*) for each experimental group/condition, given as a discrete number and unit of measurement
☐	☒	A statement on whether measurements were taken from distinct samples or whether the same sample was measured repeatedly
☐	☒	The statistical test(s) used AND whether they are one- or two-sided<br>*Only common tests should be described solely by name; describe more complex techniques in the Methods section.*
☐	☒	A description of all covariates tested
☐	☒	A description of any assumptions or corrections, such as tests of normality and adjustment for multiple comparisons
☐	☒	A full description of the statistical parameters including central tendency (e.g. means) or other basic estimates (e.g. regression coefficient) AND variation (e.g. standard deviation) or associated estimates of uncertainty (e.g. confidence intervals)
☐	☒	For null hypothesis testing, the test statistic (e.g. *F*, *t*, *r*) with confidence intervals, effect sizes, degrees of freedom and *P* value noted<br>*Give P values as exact values whenever suitable.*
☒	☐	For Bayesian analysis, information on the choice of priors and Markov chain Monte Carlo settings
☒	☐	For hierarchical and complex designs, identification of the appropriate level for tests and full reporting of outcomes
☒	☐	Estimates of effect sizes (e.g. Cohen's *d*, Pearson's *r*), indicating how they were calculated

Our web collection on statistics for biologists contains articles on many of the points above.

Software and code

Policy information about availability of computer code

Data collection	FACS Diva 8.0.1 (Flow cytometry), FCAP array v. 3.0.19 (CBA)
Data analysis	Software used to analyse the data include FlowJo version 10, Prism, R version 4.2.0 (Codes can be made available on request.), and Living Image version 4.5.5.

For manuscripts utilizing custom algorithms or software that are central to the research but not yet described in published literature, software must be made available to editors and reviewers. We strongly encourage code deposition in a community repository (e.g. GitHub). See the Nature Portfolio guidelines for submitting code & software for further information.

Data

Policy information about availability of data

All manuscripts must include a data availability statement. This statement should provide the following information, where applicable:

- Accession codes, unique identifiers, or web links for publicly available datasets
- A description of any restrictions on data availability
- For clinical datasets or third party data, please ensure that the statement adheres to our policy

The primary data supporting the results in this study are available within the paper and its Supplementary Information. Source data for tumour burden is provided with this paper. The raw and analysed datasets generated during the study are too large to be publicly shared, yet they are available for research purposes from the corresponding author on reasonable request.

Human research participants

Policy information about studies involving human research participants and Sex and Gender in Research.

Reporting on sex and gender	PBMCs and T cells from healthy donors were used. Sex and gender were not available.
Population characteristics	—
Recruitment	Healthy donors were recruited in accordance with the protocol approved by the Kyoto University School of Medicine Ethical Committee.
Ethics oversight	The use of the purchased PBMCs was approved by the Kyoto University School of Medicine Ethical Committee

Note that full information on the approval of the study protocol must also be provided in the manuscript.

Field-specific reporting

Please select the one below that is the best fit for your research. If you are not sure, read the appropriate sections before making your selection.

☒ Life sciences ☐ Behavioural & social sciences ☐ Ecological, evolutionary & environmental sciences

For a reference copy of the document with all sections, see nature.com/documents/nr-reporting-summary-flat.pdf

Life sciences study design

All studies must disclose on these points even when the disclosure is negative.

Sample size	No statistical methods were used to predetermine the experimental sample size. Sample sizes were determined on the basis of relevant literature (such as Themeli et.al, Nat. Biotech. 2013).
Data exclusions	No data were excluded.
Replication	For all figures, two or three independent experiments were performed and all attempts at replicating the observations were successful. Similar results were obtained across two laboratories.
Randomization	All samples were number-coded until the measurement was completed. For the in vivo experiments, mice were randomly assigned to each group.
Blinding	Blinding was not performed. Fully blinded experiments were not possible owing to personnel availability during the experiments.

Reporting for specific materials, systems and methods

We require information from authors about some types of materials, experimental systems and methods used in many studies. Here, indicate whether each material, system or method listed is relevant to your study. If you are not sure if a list item applies to your research, read the appropriate section before selecting a response.

Materials & experimental systems

n/a	Involved in the study	
☐	☒	Antibodies
☐	☒	Eukaryotic cell lines
☒	☐	Palaeontology and archaeology
☐	☒	Animals and other organisms
☒	☐	Clinical data
☒	☐	Dual use research of concern

Methods

n/a	Involved in the study	
☒	☐	ChIP-seq
☐	☒	Flow cytometry
☒	☐	MRI-based neuroimaging

Antibodies

Antibodies used	For T cell phenotyping, the following antibodies were used:<br>CD3-eFluor 450(clone UCHT1,eBioscience), CD3-APC-Cy7(clone UCHT1,BioLegend), CD3-APC(clone UCHT1,BioLegend), CD4-Brilliant Violet 421(clone OKT4,BioLegend), CD4-Brilliant Violet 510(clone OKT4,BioLegend), CD5-FITC(clone UCHT2,eBioscience), CD5-PE-Cy7(clone UCHT2,eBioscience), CD7-APC(clone CD7-6B7,BioLegend), CD8a-PerCP-Cy5.5(clone SK1,BioLegend), CD8b-PE-Cy7(clone

SIDI8BEE,eBioscience), CD8b-APC(clone 2ST8.5H7,BD), CD19-PE(clone HIB19,BD), CD25-FITC(clone BC96,BioLegend), CD27-APC(clone O323,BioLegend), CD28-Brilliant Violet 421(clone CD28.2,BioLegend), CD45-Brilliant Violet 510(clone HI30,BioLegend), CD45RA-Brilliant Violet 510(clone HI100,BioLegend), CD45RO-APC-Cy7(clone UCHL1,BioLegend), CD56-APC-Cy7(clone HCD56,BioLegend), CD62L-PE-Cy7(clone DREG-56,BioLegend), CD69-PacificBlue(clone FN50,BioLegend), CD94(NKG2A)-Brilliant Violet 421(clone HP-3D9,BD Biosciences), CD159a(KLRC)-PacificBlue(clone S19004C,BioLegend), CD161(KLRB)-PE-Cy7(clone HP-3G10,BioLegend), CD197(CCR7)-APC(clone G043H7,BioLegend), CD223(LAG-3)-APC-eFluor780(clone 3DS223H,eBioscience), CD226(DNAM)-APC(clone 11A8,BioLegend), CD247(pY142)-Alexa Fluor 647(clone K25-407.69,BD), CD152(CTLA-4)-APC(clone L3D10,BioLegend), CD271(NGFR)-APC-Fire750(clone ME20.4,BioLegend), CD279(PD-1)-Brilliant Violet 421(clone 29F.1A12,BioLegend), CD314(NKG2D)-PE-Cy7(clone 1D11,BioLegend), CD335(NKp46)-FITC(clone 900,BioLegend), CD336(NKp44)-APC(clone P44-8,BioLegend), CD337(NKp30)-APC(clone P30-15,BioLegend), EGFR-Brilliant Violet 421(clone AY13,BioLegend), ERK1/2 (pT202/pY204) -Alexa Fluor 647(clone 20A,BD Biosciences), Mouse IgG2a k-Alexa Fluor 647(clone MOPC-173,BD Biosciences), TCRab-FITC(clone WT31,eBioscience), TIGIT-PerCP-eFluor 710(clone MBSA43,eBioscience), Tim3-PE-Cy7(clone F38-2E2,BioLegend)

Validation

Antibodies were validated using positive and negative cells using human PBMCs or isotype controls.
Validation reports were also provided by the antibody manufacturers(BioLegend, BD biosciences and eBiosciences). Compensation controls were used for every experiment.
https://www.thermofisher.com/antibody/product/CD3-Antibody-clone-UCHT1-Monoclonal/48-0038-42
https://www.biolegend.com/en-us/search-results/apc-cyanine7-anti-human-cd3-antibody-3929
https://www.biolegend.com/en-us/products/apc-anti-human-cd3-antibody-861?GroupID=BLG5900
https://www.biolegend.com/en-us/search-results/brilliant-violet-421-anti-human-cd4-antibody-7775
https://www.biolegend.com/ja-jp/products/brilliant-violet-510-anti-human-cd4-antibody-8010
https://www.thermofisher.com/antibody/product/CD5-Antibody-clone-UCHT2-Monoclonal/11-0059-42
https://www.thermofisher.com/antibody/product/CD5-Antibody-clone-UCHT2-Monoclonal/25-0059-42
https://www.biolegend.com/en-us/products/apc-anti-human-cd7-antibody-6088
https://www.biolegend.com/en-us/products/percp-cyanine5-5-anti-human-cd8-antibody-6389?GroupID=BLG10167
https://www.thermofisher.com/antibody/product/CD8b-Antibody-clone-SIDI8BEE-Monoclonal/25-5273-42
https://www.bdbiosciences.com/en-us/products/reagents/flow-cytometry-reagents/clinical-discovery-research/single-color-antibodies-ruo-gmp/apc-mouse-anti-human-cd8.641058
https://www.bdbiosciences.com/en-ca/products/reagents/flow-cytometry-reagents/research-reagents/single-color-antibodies-ruo/pe-mouse-anti-human-cd19.555413
https://www.biolegend.com/ja-jp/products/fitc-anti-human-cd25-antibody-615
https://www.biolegend.com/en-us/products/apc-anti-human-cd27-antibody-808?GroupID=BLG7922
https://www.biolegend.com/en-us/products/brilliant-violet-421-anti-human-cd28-antibody-8170?GroupID=BLG10175
https://www.biolegend.com/ja-jp/products/brilliant-violet-510-anti-human-cd45-antibody-8006
https://www.biolegend.com/en-us/products/brilliant-violet-510-anti-human-cd45ra-antibody-8007?GroupID=GROUP658
https://www.biolegend.com/en-us/products/apc-cyanine7-anti-human-cd45ro-antibody-7372?GroupID=GROUP658
https://www.biolegend.com/en-us/products/apc-cyanine7-anti-human-cd56-ncam-antibody-7115?GroupID=BLG15664
https://www.biolegend.com/en-us/products/pe-cyanine7-anti-human-cd62l-antibody-3944
https://www.biolegend.com/en-us/products/pacific-blue-anti-human-cd69-antibody-3360?GroupID=BLG10251
https://www.bdbiosciences.com/en-us/products/reagents/flow-cytometry-reagents/research-reagents/single-color-antibodies-ruo/bv421-mouse-anti-human-cd94.743948
https://www.biolegend.com/en-us/search-results/pacific-blue-anti-human-cd159a-nkg2a-antibody-20202
https://www.biolegend.com/en-us/products/apc-cyanine7-anti-human-cd161-antibody-9972?GroupID=BLG9768
https://www.biolegend.com/en-us/products/apc-anti-human-cd197-ccr7-antibody-7536?GroupID=BLG9613
https://www.thermofisher.com/antibody/product/47-2239-42.html?ef_id=Cj0KCQjw2_OWBhDqARIsAAUNTTGJ0a5OykcKSWEwYFzjrXTkkjoIvc5IPGRGNJh5smE1GEqMQrzBGWcaAufkEALw_wcB:G:s&s_kwcid=AL!3652!3!278870232429!!!g!!&cid=bid_pca_frg_r01_co_cp1359_pjt0000_bid00000_0se_gaw_dy_pur_con&gclid=Cj0KCQjw2_OWBhDqARIsAAUNTTGJ0a5OykcKSWEwYFzjrXTkkjoIvc5IPGRGNJh5smE1GEqMQrzBGWcaAufkEALw_wcB
https://www.biolegend.com/ja-jp/products/apc-anti-human-cd226-dnam-1-antibody-8465
https://www.bdbiosciences.com/en-nz/products/reagents/flow-cytometry-reagents/research-reagents/single-color-antibodies-ruo/alexa-fluor-647-mouse-anti-cd247-py142.558489
https://www.biolegend.com/ja-jp/products/apc-anti-human-cd152-ctla-4-antibody-6999
https://www.biolegend.com/ja-jp/products/apc-fire-750-anti-human-cd271-ngfr-antibody-16306
https://www.biolegend.com/ja-jp/products/brilliant-violet-421-anti-mouse-cd279-pd-1-antibody-7330
https://www.biolegend.com/en-us/products/pe-cyanine7-anti-human-cd314-nkg2d-antibody-6499?GroupID=BLG8540
https://www.biolegend.com/en-us/products/fitc-anti-human-cd335-nkp46-antibody-8464?GroupID=BLG8494
https://www.biolegend.com/en-us/search-results/apc-anti-human-cd336-nkp44-antibody-3850
https://www.biolegend.com/en-us/products/apc-anti-human-cd337-nkp30-antibody-3856?GroupID=BLG5091
https://www.biolegend.com/en-us/products/brilliant-violet-421-anti-human-egfr-antibody-8621
https://www.bdbiosciences.com/en-de/products/reagents/flow-cytometry-reagents/research-reagents/single-color-antibodies-ruo/alexa-fluor-647-mouse-igg2a-isotype-control.558053
https://www.bdbiosciences.com/en-us/products/reagents/flow-cytometry-reagents/research-reagents/single-color-antibodies-ruo/alexa-fluor-647-mouse-anti-erk1-2-pt202-py204.561992
https://www.thermofisher.com/antibody/product/TCR-alpha-beta-Antibody-clone-WT31-Monoclonal/11-9955-42
https://www.thermofisher.com/antibody/product/TIGIT-Antibody-clone-MBSA43-Monoclonal/46-9500-42
https://www.biolegend.com/ja-jp/products/pe-cyanine7-anti-human-cd366-tim-3-antibody-8303

Eukaryotic cell lines

Policy information about cell lines and Sex and Gender in Research

Cell line source(s)

TKT3V1-7 is an iPS cell line established by Nishimura et. al (Cell Stem Cell, 2013).
HepG2(JCRB1054) ,JHH7(JCRB1031) and Nalm6(CRL-3273) were purchased from JCRB.
SK-Hep1-GPC3, SK-Hep1-Vector, K562, RD-18 and KOC7c were provided by co-authors.

Authentication	The expression of glypican3 was checked by flow-cytometric analysis.
Mycoplasma contamination	All cell lines were confirmed negative for mycoplasma contamination.
Commonly misidentified lines (See ICLAC register)	None of the cell lines used in the study are listed in the ICLAC Database of Cross-contaminated or Misidentified Cell Lines.

Animals and other research organisms

Policy information about studies involving animals; ARRIVE guidelines recommended for reporting animal research, and Sex and Gender in Research

Laboratory animals	6–12 week-old female NOD-SCID IL2Rγcnull (NSG) mice were purchased from Oriental Bio (Yokohama, Japan). Mice were exposed to 12h:12 h light–dark cycles with free access to water and food. The ambient temperature was restricted to 20–26 degrees Celsius and room humidity was 40–70%.
Wild animals	The study did not involve wild animals.
Reporting on sex	Female mice were used.
Field-collected samples	The study did not involve samples collected from the field.
Ethics oversight	All animal experiments were performed in accordance with the Kyoto University School of Medicine Ethical Committee.

Note that full information on the approval of the study protocol must also be provided in the manuscript.

Flow Cytometry

Plots

Confirm that:

- [x] The axis labels state the marker and fluorochrome used (e.g. CD4-FITC).
- [x] The axis scales are clearly visible. Include numbers along axes only for bottom left plot of group (a 'group' is an analysis of identical markers).
- [x] All plots are contour plots with outliers or pseudocolor plots.
- [x] A numerical value for number of cells or percentage (with statistics) is provided.

Methodology

Sample preparation	All stains were performed with < 1x10^6 cells per 100 µL staining buffer (PBS + 2% FBS) with 1:100 dilution of each antibody, 20 min on ice in dark.
Instrument	Stained cell samples were analyzed using LSR or FACS AriaII flow cytometer (BD Biosciences).
Software	The data were processed using FlowJo (Tree Star).
Cell population abundance	Sorted samples were confirmed for purity post-sort via flow cytometry. Sorted populations were confirmed to be of >95% purity.
Gating strategy	All human cells were first gated on FSC/SSC according to cell size and granularity, using stained human peripheral mononuclear cells (PBMCs) as a positive control and reference for cell size, granularity and staining intensity. Unstained samples were used to set up negative gates, and stained human PBMCs were used to set up positive gates. Dead-cell populations were excluded using PI staining.

- [ ] Tick this box to confirm that a figure exemplifying the gating strategy is provided in the Supplementary Information.
