# Supplementary figures and images for: Optimization of the proliferation and persistency of CAR T cells derived from human induced pluripotent stem cells
Source: Nat Biomed Eng. 2022 Dec 12;7(1):24–37. doi: 10.1038/s41551-022-00969-0 (PMC9870784; doi:10.1038/s41551-022-00969-0)

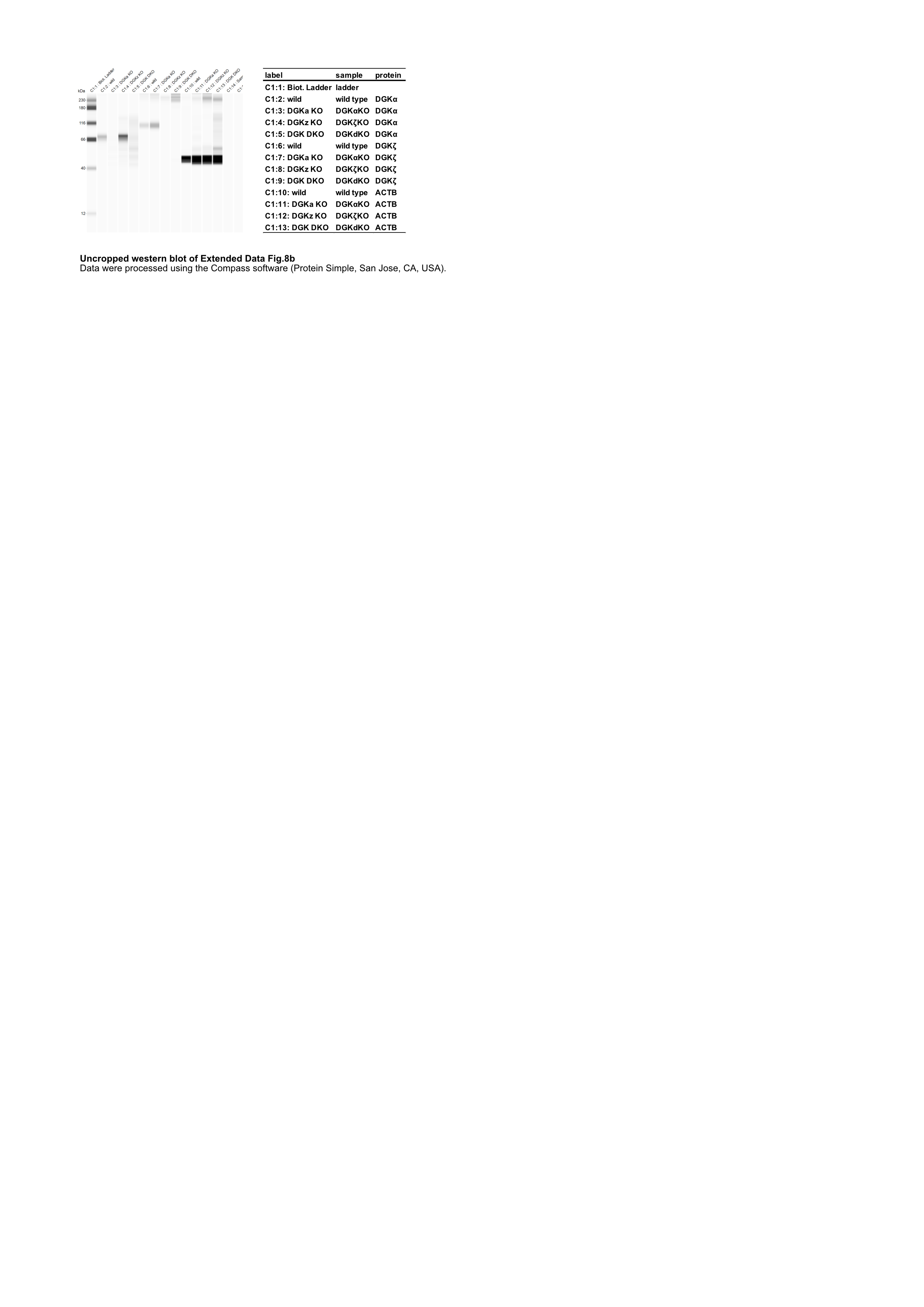

Supplement: Source Data for Extended Data Fig. 8 — Uncropped western blots for Extended Data Fig. 8b. [file 41551_2022_969_MOESM6_ESM.tiff]
